# A multiplex real-time fluorescence-based quantitative PCR assay for calf diarrhea viruses

**DOI:** 10.3389/fmicb.2023.1327291

**Published:** 2024-01-05

**Authors:** Wenxin Meng, Zihan Chen, Qifeng Jiang, Jinping Chen, Xiaoying Guo, Zihang Ma, Kun Jia, Shoujun Li

**Affiliations:** ^1^College of Veterinary Medicine, South China Agricultural University, Guangzhou, China; ^2^Guangdong Technological Engineering Research Center for Pet, Guangzhou, China

**Keywords:** multiplex real-time fluorescent quantitative PCR, bovine torovirus, bovine enterovirus, bovine norovirus, bovine coronavirus, bovine rotavirus, bovine viral diarrhea virus

## Abstract

**Introduction:**

Calf diarrhea is a significant condition that has a strong effect on the cattle industry, resulting in huge economic losses annually. Bovine torovirus (BToV), bovine enterovirus (BEV), bovine norovirus (BNoV), bovine coronavirus (BCoV), bovine rotavirus (BRV), and bovine viral diarrhea virus (BVDV) are key pathogens that have been implicated in calf diarrhea. Among these viruses, there remains limited research on BToV, BEV, and BNoV, with no available vaccines or drugs for their prevention and control. Although commercial vaccines exist for BCoV, BRV, and BVDV, the prevalence of these diseases remains high.

**Methods:**

To address this issue, we developed a multiplex real-time fluorescence quantitative PCR method for detecting BToV, BEV, BNoV, BCoV, BRV, and BVDV. This method can be used to effectively monitor the prevalence of these six viruses and serve as a reference for future prevention and control strategies. In this study, we specifically designed primers and probes for the BNoV Rdrp, BEV 5′UTR, BToV M, BCoV N, BRV NSP5, and BVDV 5′UTR genes.

**Results:**

This method was determined to be efficient, stable, and sensitive. The lowest detectable levels of plasmids for BNoV, BEV, BToV, BRV, BCoV, and BVDV were 1.91 copies/μL, 96.0 copies/μL, 12.8 copies/μL, 16.4 copies/μL, 18.2 copies/μL, and 65.3 copies/μL, respectively. Moreover, the coefficients of variation for all six detection methods were < 3%; they also exhibited a strong linear relationship (R^2^ ≥ 0.98), and an amplification efficiency of 90%−110%. A total of 295 fecal and anal swabs were collected from calves with diarrhea in Guangdong, China. The positive rates for BToV, BEV, BNoV, BCoV, BR, and BVDV were determined to be 0.34% (1/295), 6.10% (18/295), 0.68% (2/295), 1.36% (4/295), 10.85% (32/295), and 2.03% (6/295), respectively. Notably, BEV and BRV exhibited the highest prevalence.

**Discussion:**

Additionally, this study identified the occurrence of BToV and BNoV in Guangdong for the first time. In summary, this study successfully established an effective method for detecting several important bovine viruses; ultimately, this holds strong implications for the future development of the cattle industry.

## 1 Introduction

Calf diarrhea is a significant condition affecting the global cattle industry. Various etiological agents have been associated with the development of calf diarrhea, including parasites, bacteria, and viruses.

Toroviruses (Nidovirales, Tornidovirineae, Tobaniviridae, Torovirinae, and *Torovirus*) are single-stranded positive-sense RNA viruses that target the digestive tract. These viruses have been detected in several species, including cattle, pigs, horses, goats, and humans (Hoet and Saif, 2004; Hu et al., 2019). Bovine toroviruses (BToVs) were first identified during a diarrhea outbreak in the United States in 1982 (Woode et al., 1982). Specifically, a high prevalence of BToV infection has been observed among 3 week-old calves with diarrhea (Hoet et al., 2003). Currently, BToV has been reported in 17 countries worldwide, which has attracted increasing attention (Zhao et al., 2021).

Bovine enterovirus (BEV; Picornavirales, Picornaviridae, Ensavirinae, and *Enterovirus*) is a non-enveloped single-stranded positive-sense RNA virus that belongs to the *Enterovirus* genus (Beato et al., 2018). Initially, this virus was considered non-pathogenic. However, recent studies have shown that, although most infected cattle exhibit subclinical symptoms (Palacios and Oberste, 2005), several isolated BEV strains have been determined to cause severe clinical symptoms (Blas-Machado et al., 2011; Zhang et al., 2014), including diarrhea, bloody stools, respiratory issues, reduced milk production, and reproductive disorders, ultimately resulting in substantial economic losses (Zhu et al., 2014).

Bovine norovirus (BNoV; Picornavirales, Caliciviridae, and *Norovirus*), a member of the Caliciviridae family within the *Norovirus* genus, has been characterized by its single-stranded positive-sense RNA genome and lack of a viral envelope (Hardy, 2005). Noroviruses are a significant cause of severe diarrhea in several species, including pigs, cattle, sheep, dogs, cats, mice, and humans (Villabruna et al., 2019). BNoV infections were first reported in the United Kingdom in 1976, but have been subsequently reported in various countries, including the United States, Germany, Italy, South Korea, and China (Bhella and Goodfellow, 2011). Regression tests have confirmed that BNoV infections can cause severe diarrhea and intestinal lesions (Otto et al., 2011; Jung et al., 2014). This virus primarily causes diarrhea in calves but can also be detected in the feces of adult heifers and asymptomatic calves.

Bovine coronavirus (BCoV; Nidovirales, Cornidovirineae, Coronaviridae, Orthocoronavirinae, and *Betacoronavirus*). It is a single-stranded positive-sense RNA virus that can induce diarrhea in newborn calves, winter dysentery in adult cattle, and respiratory diseases in both newborn and adult cattle (Bartels et al., 2010; Saif, 2010). Bovine coronaviruses were first identified and isolated in 1973 (Mebus et al., 1973) and were subsequently detected globally (Zhu et al., 2022).

Bovine rotavirus (BRV; Resentoviricetes, Reovirales, Sedoreoviridae, and *Rotavirus*), comprises seven distinct groups (A–G). Group A rotaviruses are primarily responsible for diarrhea in calves, with calves under 7 days old being the most susceptible group. Overall, BRV infection predominantly affects calves aged 15–45 days, and can lead to symptoms such as diarrhea, dehydration, and depression (Wang et al., 2019). It is also associated with high rates of infection and mortality in these calves (Chen et al., 2022; Qin et al., 2022).

Bovine viral diarrhea virus (BVDV; Flaviviridae and *Pestivirus*) is an enveloped single-stranded positive-sense RNA virus (Peterhans et al., 2010). It was first identified and isolated in the United States in 1946, but has since been reported in numerous countries worldwide (Iqbal et al., 2004; Scharnböck et al., 2018). BVDV is a significant infectious disease affecting the cattle industry by causing immune disorders and hemorrhagic infections; ultimately, this may result in severe gastrointestinal mucosal erosion, necrosis, gastroenteritis, and diarrhea. In particular, BVDV infection of pregnant cows can lead to reproductive disorders such as decreased milk production, abortion, fetal growth retardation, and fetal malformation (Lanyon et al., 2014). Although BVDV can infect cattle of all ages, calves are particularly vulnerable.

All six of these viruses can induce varying degrees of diarrheal symptoms in calves, with BToV, BEV, BCoV, and BVDV also causing respiratory symptoms. Co-infection of these viruses with other viruses or bacteria complicates diagnosis, making targeted prevention and control measures challenging. Presently, different detection methods have been established to detect the above six pathogens; however, there is no rapid simultaneous detection method (Wang et al., 2019; Chen et al., 2023; Han and Han, 2023). In particular, there are few studies on the detection methods related to BNoV, and the virus can cause severe diarrhea in calves, so the prevention and control of BNoV is relatively difficult, which needs us to pay enough attention. Therefore, there is an urgent need to establish a rapid, sensitive, and stable method for detecting these six viruses. Therefore, in this study, we aimed to establish a six-pair real-time fluorescence quantitative PCR method using the conserved sequences of these six viruses to design specific primers and probes. Moreover, we implemented two reaction systems using the same reaction program to simultaneously detect these viruses, significantly reducing detection time.

## 2 Materials and methods

### 2.1 Primer and probe design

To design specific primers and probes, we aligned 9, 15, 10, 32, and 27 sequences of BToV, BEV, BnoV, BcoV, BRV, and BVDV strains, respectively, collected from the National Center for Biotechnology Information (NCBI) database and identified the most conserved fragments within these sequences. After conducting sequence alignment, the following regions were selected for these primers and probes: the 5′UTR gene of BEV, the M gene of BToV, the RDRP gene of BNoV, the N gene of BCoV, the NSP5 gene of BRV, and the 5′UTR gene of BVDV (Table 1) (GenBank accession numbers are:MN882587.1, MK639928.1, MK639928.1, ON142320.1, JN831208.1, and MN417910.1, respectively). The designed primers and probes were analyzed for specificity using BLAST (NCBI). All primers and probes were synthesized by Shanghai Sangon Biological (Shanghai, China).

**Table 1:** Primer and probe information.

**Target**	**Gene**	**Sequence (5^′^to 3^′^)**	**Size (bp)**
BEV	5′UTR	P1: ATGCTGCTAATCCCAACCTC	97
		P2: GRGTACCGAAAGTAGTCTGTTC	
		Probe: VIC-ACAAHCCAGTGTTGCTRCGTCGTAAYG-MGB	
BToV-R	M	P1: TATATGGCGCGGTTTGGAG	104
		P2: AGGCCCAATARCCWGTAAAG	
		Probe: CY5-ATWGCCTATTGGTGGCTTCCCAGTATG-MGB	
BNoV-R	Rdrp	P1: CAGGTGATGGACCARCCMAA	114
		P2: GCCATTCCARTCRTCATTCT	
		Probe: FAM-TCTTGTCCAGRGACATGCARGCRTCC-MGB	
BCoV	N	P1: AGAACCCCTACCTCTGGTGT	156
		P2: CTTCTGGCGGGGCTTATTC	
		Probe: VIC-CAAGGATGCCACTAAGCCACAG-MGB	
BRV	NSP5	P1: AACGATCCACTCACCAGCTTT	105
		P2: ATTGCTTGATGGTCGTGATTG	
		Probe: FAM-TGAATCCATAGACACGCCAGC-MGB	
BVDV	5′UTR	P1: CATGCCCTTAGTAGGACTAGC	99
		P2: CGAACCACTGACGACTACC	
		Probe: CY5-AGCCATCCAAYGAACTCRCCAC-MGB	

### 2.2 Sample preparation

Sample RNA was extracted using the RNAfast200 kit (Shanghai Feijie Bio-Technology Co., Ltd., Shanghai, China). PBS solution (1 mL) was added to 10 mg feces or anal swabs and mixed thoroughly. Samples were then centrifuged at 12000 rpm at 4°C for 1 min. The supernatant was removed and set aside. Lysate (500 μL) was added, the mixture placed upside down, and allowed to stand for 1 min. The lysed samples were transferred to an inner cannula and centrifuged at 12000 rpm for 1 min at 4°C. The liquid was discarded, wash solution (500 μL) added to the inner cannula, the mixture was centrifuged for 1 min at 12000 rpm and 4°C, the liquid discarded, and the procedure was repeated. After the final liquid discarding step, the samples were centrifuged at 12000 rpm for 1 min at 4°C. The inner cannula was transferred to a clean EP tube and allowed to sit for 3–5 min. After the liquid was completely evaporated, 25–50 μL elution buffer was added to the center of the membrane, left at room temperature for 1 min, centrifuged at 12000 rpm at 4°C for 1 min, and sample RNA was obtained. RNA quality was assessed using a Nano Drop One ultramicrospectrophotometer (Thermo Fisher Scientific., Shanghai, China), and stored at −80°C until further use. RNA was reverse-transcribed to cDNA using HiScript III All-in-one RT SuperMix (Nanjing Vazyme Biotech Co., Ltd., Nanjing, China) and stored at −20°C until use.

### 2.3 Preparation of standard plasmids

The amplification products of the M gene from BToV, 5′UTR gene from BEV, Rdrp gene from BNoV, N gene from BCoV, NSP5 gene from BRV, and 5′UTR gene from BVDV were ligated into the pMD18-T vector (Takara Biotechnology (Dalian) Co., Ltd., Dalian, China) and transformed into DH-5α (Guangzhou Xinkailai Biotechnology Co., Ltd., Guangzhou, China) competent cells. Single-positive colonies were selected, cultured, and sent to Shanghai Sangon Biotechnology for sequencing. The accurately sequenced plasmids were named pMD18-T-BToV, pMD18-T-BEV, pMD18-T-BNoV, pMD18-T-BCoV, pMD18-T-BRV, and pMD18-T-BVDV. Finally, DNA was extracted from the transformed cells using an EndoFree Mini Plasmid Kit II (Tiagen Biochemical Technology, Beijing, China), and stored at −20°C for subsequent experiments.

### 2.4 Reaction system procedure and optimization

In this study, BEV, BToV, and BNoV were used in reaction system 1; BCoV, BRV, and BVDV were used in reaction system 2. Both amplification systems followed the same reaction procedure. Each reaction system was prepared to a total volume of 20 μL, comprising 10 μL of AceQ qPCR Probe Master mix (Nanjing Vazyme Biotech Co., Ltd., Nanjing, China) and 2 μL of DNA template, alongside variable volumes of the three pairs of primers, three probes, and sterile double-distilled water. Primer and probe concentrations were optimized using 10^6^ copies/μL of BEV, BToV, BNoV, BCoV, BRV, and BVDV standard plasmids as templates. A LightCycler 480 II real-time fluorescent quantitative PCR instrument (Roche Diagnostics (Shanghai) Limited, Shanghai, China) was used for amplification and analysis. The reaction program was conducted as follows: 95°C for 5 min, followed by 45 cycles of 95°C for 10 s and 60°C for 30 s. Fluorescence signals were detected at 60°C. Furthermore, we optimized the annealing temperature by testing six gradients ranging from 57°C to 62°C.

### 2.5 Establishing a standard curve

To construct standard curves, we performed 10-fold dilutions of the standard plasmid DNA samples from the six viruses, resulting in a range of concentrations from 10^8^ copies/μL to 10^2^ copies/μL. Multiple dilutions of plasmid DNA were conducted sequentially for amplification, according to the optimized reaction systems and procedures. The standard curve for multiplex real-time fluorescence quantitative PCR was established by plotting the logarithm of the initial template number of the standard plasmid DNA as the X-axis and the Ct corresponding multiplex real-time fluorescence quantitative PCR as the Y-axis. Images were drawn using GraphPad Prism 8 (GraphPad Software, California, United States).

### 2.6 Sensitivity test

The standard plasmid DNA samples from the six viruses were diluted 10-fold to concentrations ranging from 10^5^ copies/μL to 10^0^ copies/μL of standard plasmid DNA. The sensitivity of our assay was determined by testing various dilutions of plasmid DNA with this optimized reaction system; double-distilled water was used as a negative control. Images were drawn using GraphPad Prism 8.

### 2.7 Specificity test

The specificity of our multiplex real-time PCR assay was evaluated using the optimized reaction system. Here, we used cDNA or DNA from BEV, BToV, BNoV, BCoV, BRV, BVDV, bovine ephemeral fever virus (BEFV), lump skin disease virus (LSDV), and bovine respiratory syncytial virus (BRSV) as templates; sterile double-distilled water was used as a negative control. All templates were verified using PCR or viral isolation methods. Images were drawn using GraphPad Prism 8.

### 2.8 Repeatability test

To evaluate the repeatability of our assay, we prepared standard plasmid DNA from the six viruses, diluting them 10-fold; then, 10^7^ copies/μL, 10^6^ copies/μL, and 10^5^ copies/μL plasmid DNA were used as templates. Three intra- and inter-assay tests were performed using the optimized reaction system and reaction conditions to calculate the average Ct, standard deviation (SD), and coefficient of variation (CV). Finally, the repeatability and stability of the method were analyzed according to the CV calculation results.

### 2.9 Sample testing

Finally, we collected a total of 295 samples from Guangdong, China, consisting of fecal and anal swabs obtained from calves with diarrhea. RNA was extracted using RNAfast200 and reverse transcribed into cDNA using HiScript III All-in-one RT SuperMix for future experiments. These samples were then tested using the method established in this study.

## 3 Results

### 3.1 Preparation of standard plasmids

Standard plasmids were extracted and their corresponding concentrations were determined. Finally, their copy numbers were calculated according to the following formula:


$$\begin{array}{l} Copy\;number\;\left(copies/\mu L\right)\\ =\frac{Concentration\;\left(ng/\mu L\right)\;\times \;10^{-9}\;\times \;6.022\;\times \;10^{23}}{DNA\;length\;\times \;660}\; \end{array}$$

**Figure 1:**
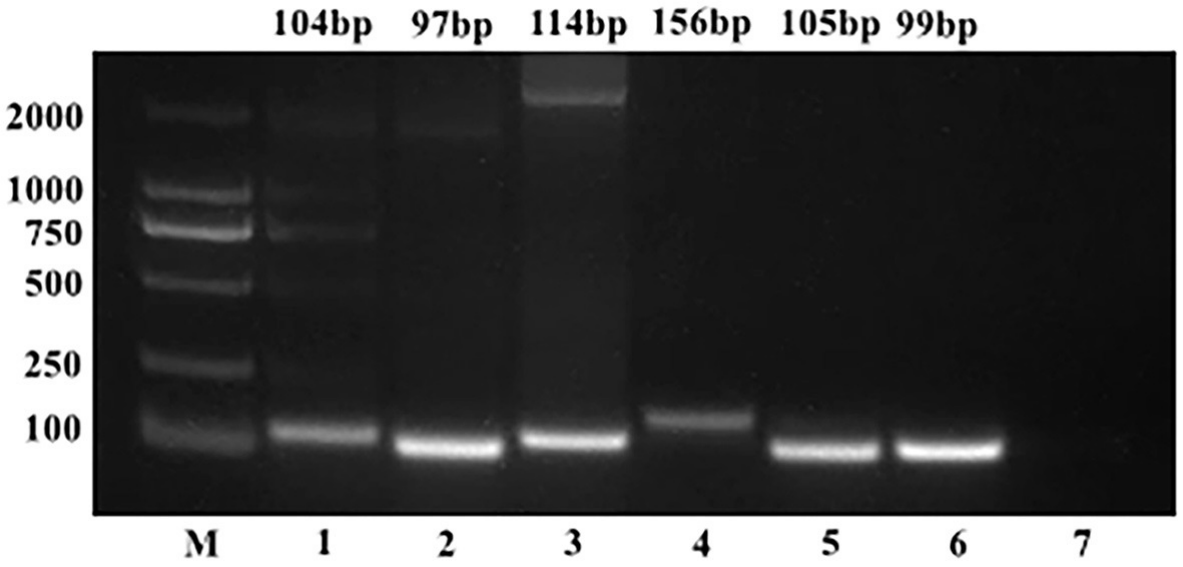
PCR results of standard plasmids from six viruses. M, DL2000 Marker; (1) BToV standard plasmid; (2) BEV standard plasmid; (3) BNoV standard plasmid; (4) BCoV standard plasmid; (5) BRV standard plasmid; (6) BVDV standard plasmid; (7) negative control.

**Figure 2:**
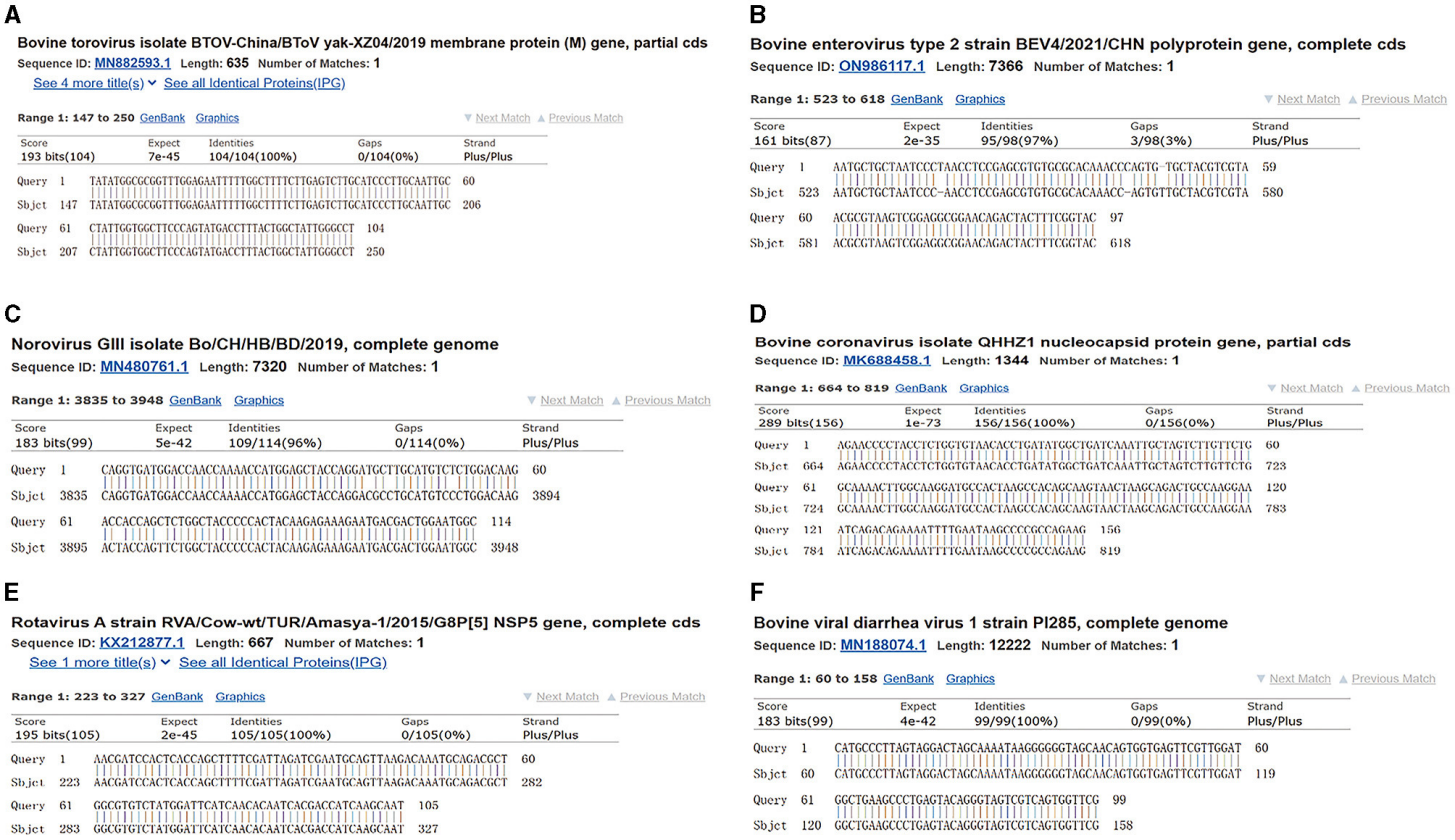
Plasmid sequence alignment results of six viruses. **(A)** BToV standard plasmid; **(B)** BEV standard plasmid; **(C)** BNoV standard plasmid; **(D)** BCoV standard plasmid; **(E)** BRV standard plasmid; **(F)** BVDV standard plasmid.

The copy numbers of the standard plasmids were determined to be 1.28 × 10^11^ copies/μL, 9.60 × 10^10^ copies/μL, 1.91 × 10^11^ copies/μL, 1.82 × 10^10^ copies/μL, 1.64 × 10^10^ copies/μL, and 6.53 × 10^10^ copies/μL for BToV, BEV, BNoV, BCoV, BRV, and BVDV, respectively. Moreover, sequencing results confirmed the presence of these genomes (Figures 1, 2).

### 3.2 Reaction system procedure and optimization

In this study, we established two optimal reaction systems, which are detailed in Tables 2, 3. The optimal annealing temperature for both reaction systems was 60°C.

**Table 2:** PCR reaction system 1.

**Reaction components**	**Reaction system (20 μL)**
2 × Probe master mix	10 μL
BToV	P1: 0.4 μL (10 μmol/L)
	P2: 0.4 μL (10 μmol/L)
	Probe: 0.4 μL (10 μmol/L)
BEV	P1: 0.5 μL (10 μmol/L)
	P2: 0.5 μL (10 μmol/L)
	Probe: 0.3 μL (10 μmol/L)
BNoV	P1: 0.5 μL (10 μmol/L)
	P2: 0.5 μL (10 μmol/L)
	Probe: 0.3 μL (10 μmol/L)
DNA template	2 μL
ddH_2_O	4.3 μL

**Table 3:** PCR reaction system 2.

**Reaction components**	**Reaction system (20 μL)**
2 × Probe master mix	10 μL
BCoV	P1: 0.3 μL (10 μmol/L)
	P2: 0.3 μL (10 μmol/L)
	Probe: 0.3 μL (10 μmol/L)
BRV	P1: 0.4 μL (10 μmol/L)
	P2: 0.4 μL (10 μmol/L)
	Probe: 0.3 μL (10 μmol/L)
BVDV	P1: 0.6 μL (10 μmol/L)
	P2: 0.6 μL (10 μmol/L)
	Probe: 0.2 μL (10 μmol/L)
DNA template	2 μL
ddH_2_O	4.6 μL

### 3.3 Standard curve and sensitivity analysis

**Figure 3:**
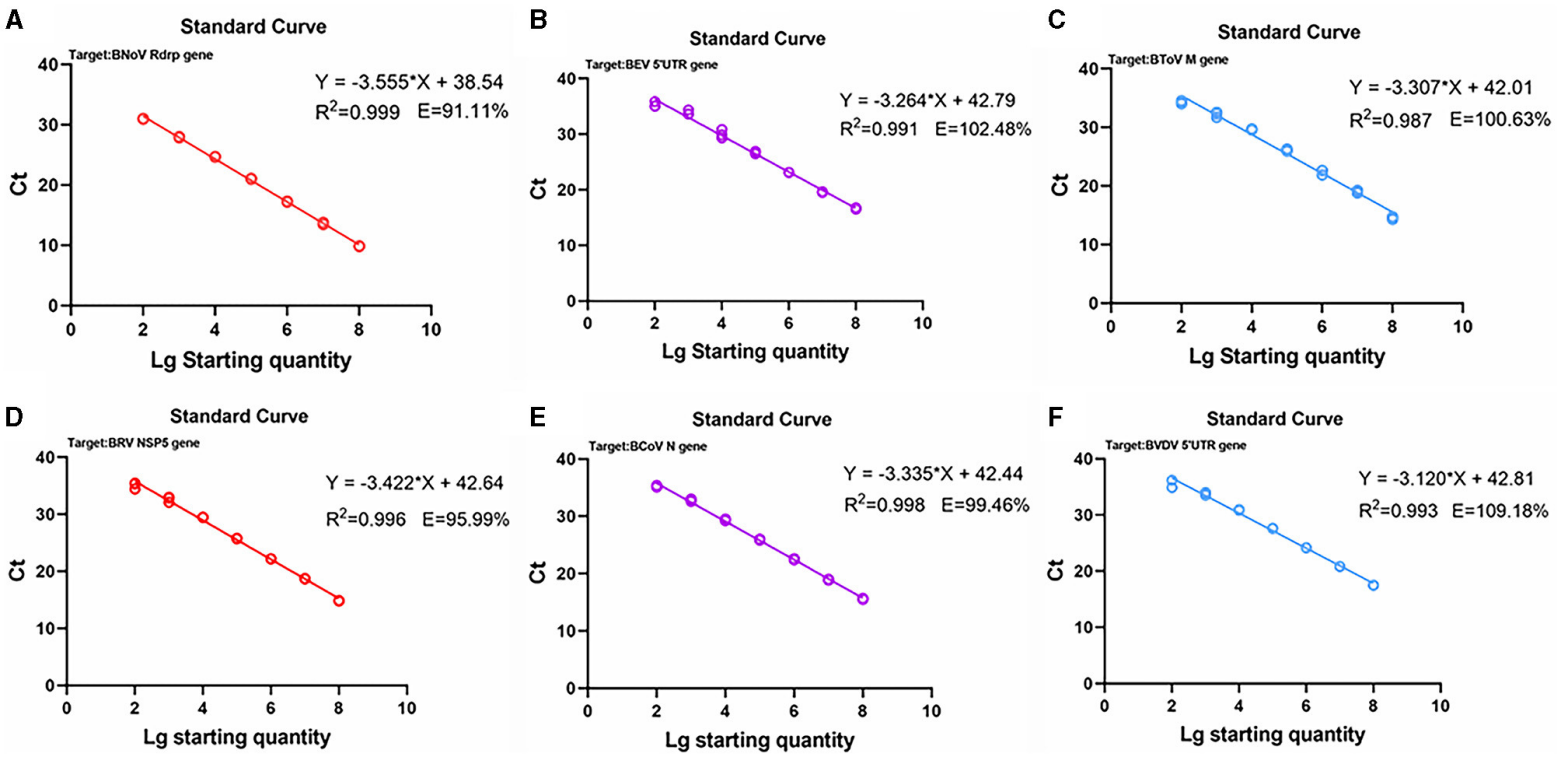
Standard curves based on the PCR amplification of six viral plasmids. **(A)** BNoV; **(B)** BEV; **(C)** BToV; **(D)** BRV; **(E)** BCoV; **(F)** BVDV.

**Figure 4:**
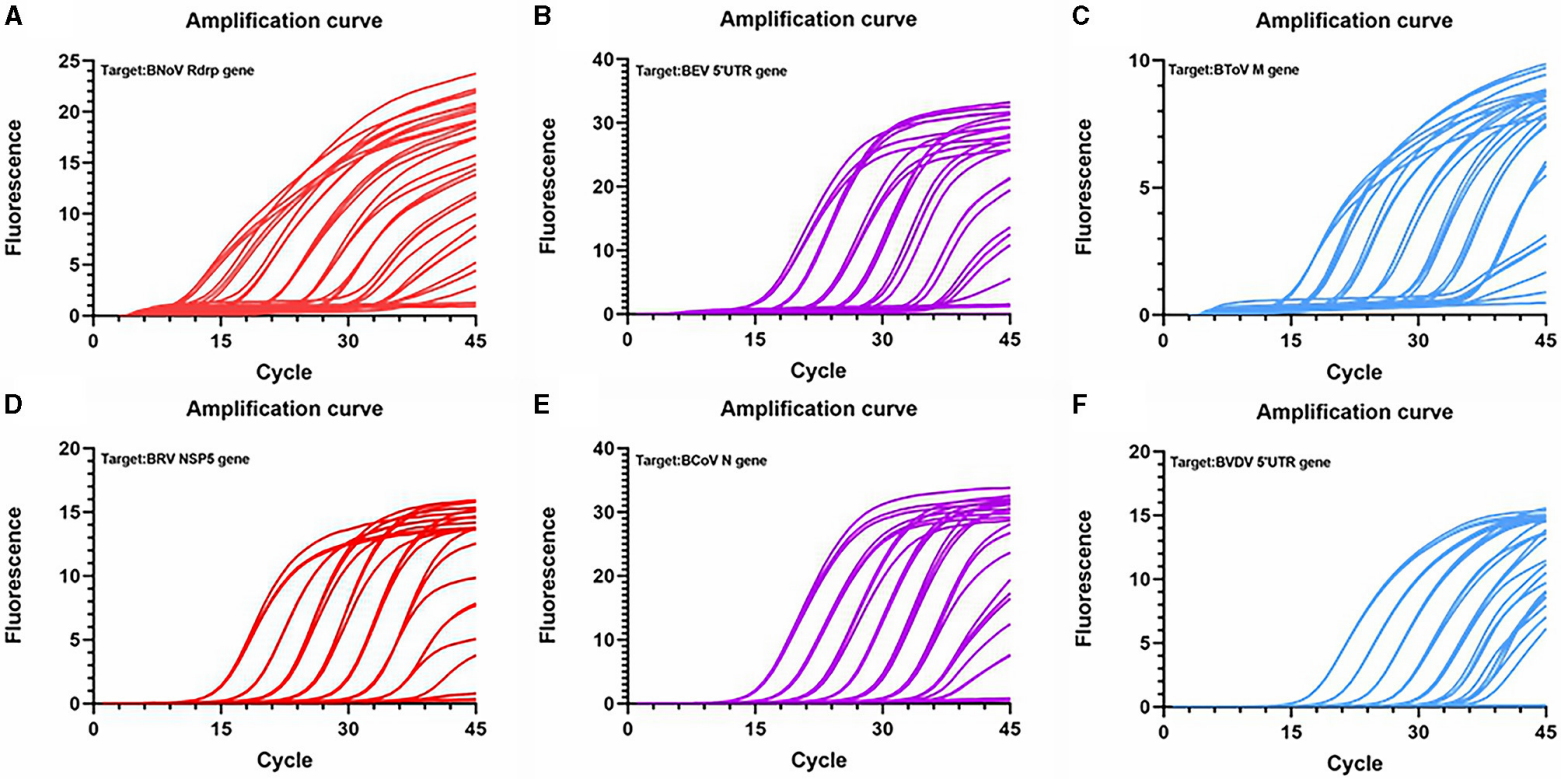
Amplification curve based on the PCR amplification of six viral plasmids. **(A)** BNoV; **(B)** BEV; **(C)** BCoV; **(D)** BRV; **(E)** BcoV; **(F)** BVDV.

Next, standard curves were established based on the amplification results, which utilized 10^2^ to 10^8^ copies/μL diluted standard plasmid DNA (Figures 3, 4). Specifically, a linear relationship was established between the lg starting quantity (X axis) and the Ct (Y axis). These relationships were as follows: BNoV expression, Y = −3.555X + 38.54, E = 91.11%, R^2^ = 0.999; BEV expression, Y = −3.264X + 42.79, E = 102.48%, R^2^ = 0.991; BToV expression, Y = −3.307X + 42.01, E = 100.63%, R^2^ = 0.987; BRV expression, Y = −3.422X + 42.64, E = 95.99%, R^2^ = 0.996; BCoV expression, Y = −3.335X + 42.44, E = 99.46%, R^2^ = 0.998; and BVDV expression, Y = −3.120X + 42.81, E = 109.18%, R^2^ = 0.993.

**Figure 5:**
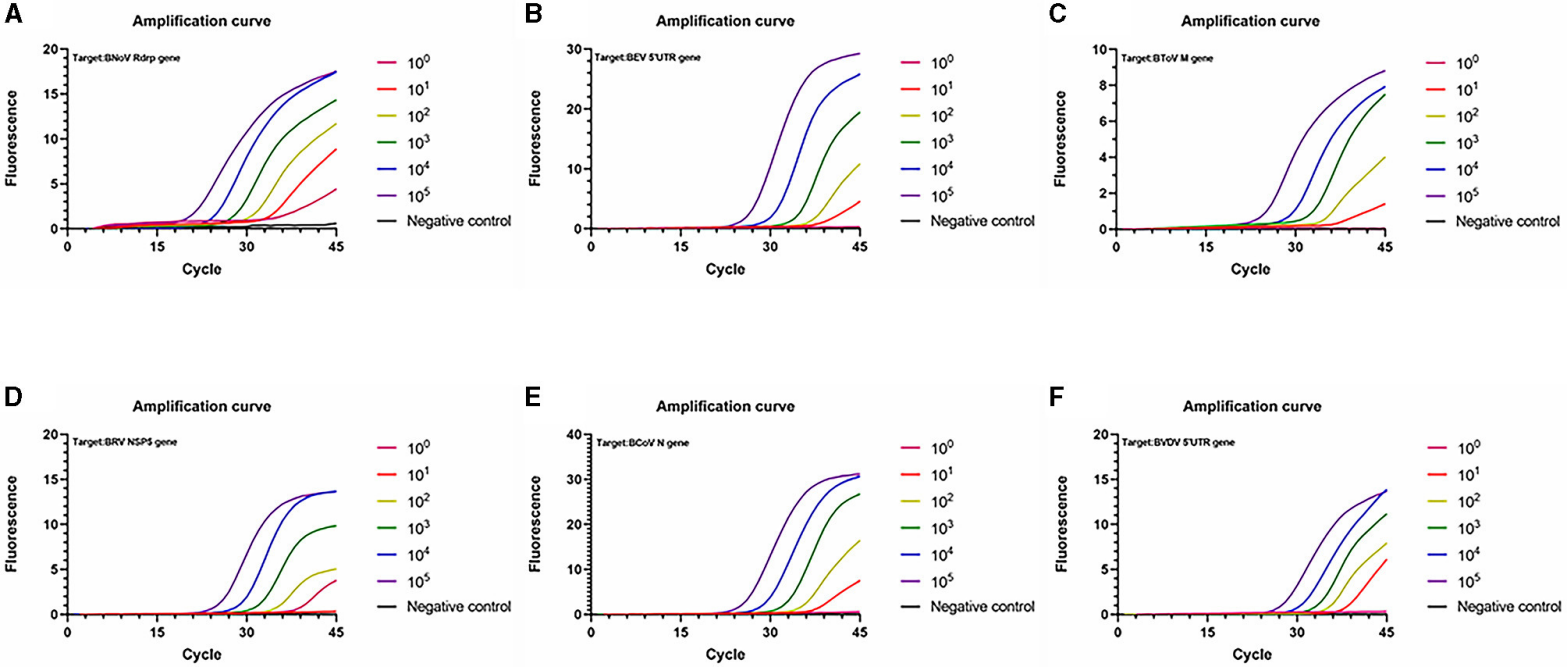
Amplification curve showing the sensitivity of the PCR amplification of six viruses. **(A)** BNoV; **(B)** BEV; **(C)** BToV; **(D)** BRV; **(E)** BCoV; **(F)** BVDV.

**Figure 6:**
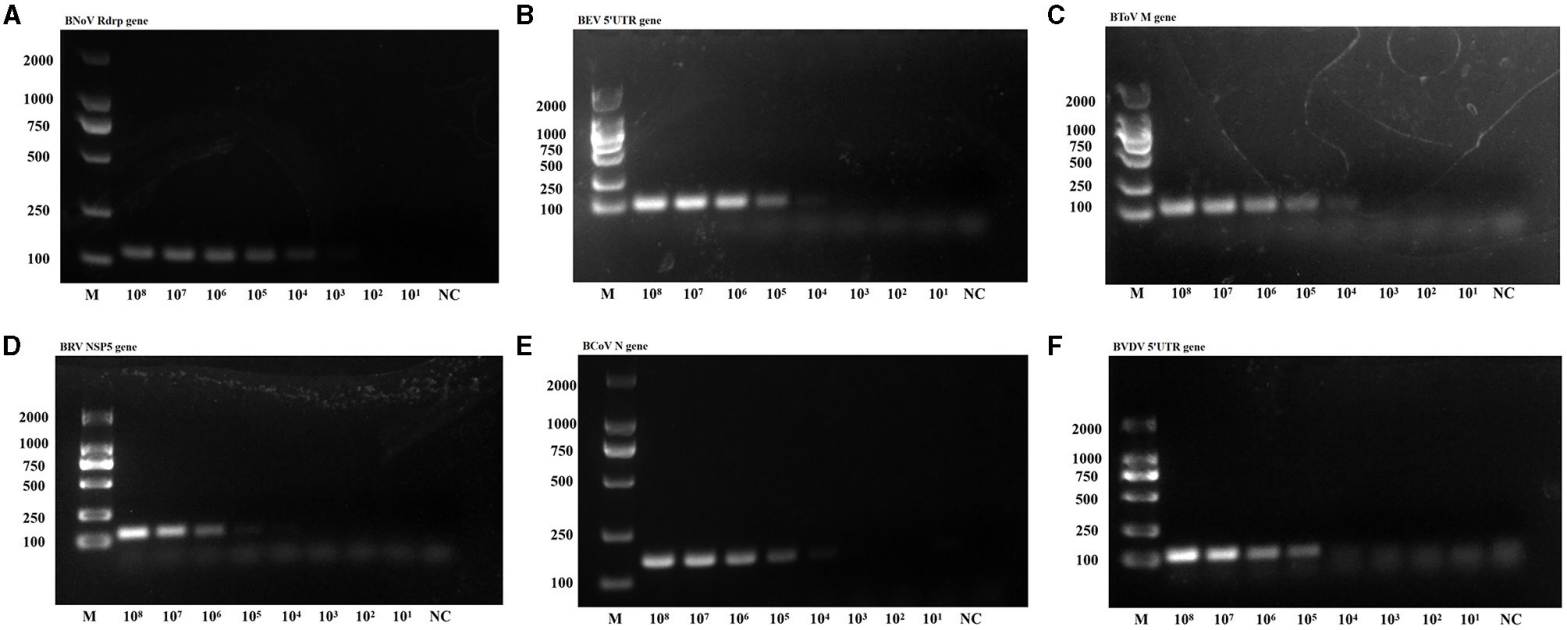
PCR sensitivity of the six viruses. **(A)** BNoV; **(B)** BEV; **(C)** BToV; **(D)** BRV; **(E)** BCoV; **(F)** BVDV.

Sensitivity tests were then conducted using the plasmids from the six viruses as templates (Figure 5). The lowest detectable levels of BNoV, BEV, BToV, BRV, BCoV, and BVDV were determined to be 1.91 copies/μL, 96.0 copies/μL, 12.8 copies/μL, 16.4 copies/μL, 18.2 copies/μL, and 65.3 copies/μL, respectively. Conversely, none of the negative controls exhibited amplification. The detection limits of traditional PCR for BNoV, BEV, BToV, BRV, BCoV and BVDV were 1.91 × 10^4^ copies/μL, 9.6 × 10^4^ copies/μL, 1.28 × 10^4^ copies/μL, 1.64 × 10^5^ copies/μL, 1.82 × 10^4^ copies/μL, and 6.53 × 10^5^ copies/μL, respectively (Figure 6). Compared with traditional PCR, the sensitivity of our system was increased by 1000–10000 times.

### 3.4 Specificity analysis

**Figure 7:**
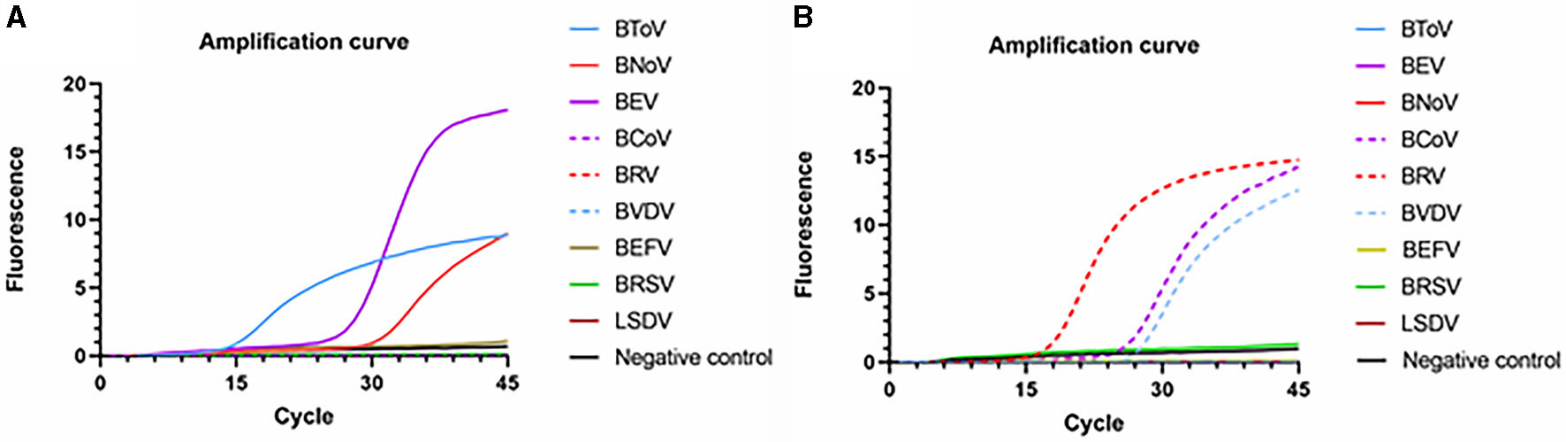
Specific amplification curves based on the PCR amplification of six viral plasmids. **(A)** Reaction system 1; **(B)** Reaction system 2.

Using identical reaction procedures, different cDNAs and DNAs were added to the two amplification systems (Figure 7). In reaction system 1, only BToV, BEV, and BNoV exhibited fluorescence signals; moreover, the amplification curve was determined to be acceptable. Similarly, in reaction system 2, only BCoV, BRV, and BVDV resulted in the detection of fluorescence signals. Neither fluorescence signals nor an amplification curve was observed in the wells containing interfering DNA or negative controls. These findings demonstrate that this method has good specificity, with no non-specific amplification.

### 3.5 Reproducibility analysis

Next, three intra- and inter-assays were performed with differing concentrations of the six plasmids (Table 4). Overall, the CVs for BToV, BEV, BNoV, BCoV, BRV, and BVDV ranged between 0.55%−1.37%, 0.44%−2.38%, 0.25%−1.91%, 0.11%−2.27%, 0.01%−0.32%, and 0.02%−0.20%, respectively. All CVs were <3%, indicating that this multiplex real-time fluorescence quantitative PCR method had good repeatability and stability. The CV was determined according to the following equation:


$$\begin{array}{l} Coefficent\;of\;variation=\frac{Standard\;Deviation\;\left(SD\right)}{Mean\;\left(MN\right)}\times 100\; \end{array}$$

**Table 4:** Reproducibility of the multiplex real-time fluorescence quantitative PCR method established in this study.

**Target**	**Plasmid concentration (copies/μL)**	**Intra-assay**			**Inter-assay**		
		**MN**	**SD**	**CV (%)**	**MN**	**SD**	**CV (%)**
BToV	10^7^	19.97	0.11	0.55	19.83	0.11	0.58
	10^6^	23.59	0.19	0.79	23.44	0.13	0.58
	10^5^	27.64	0.38	1.37	27.17	0.33	1.22
BEV	10^7^	20.70	0.09	0.44	20.56	0.16	0.76
	10^6^	24.14	0.30	1.23	24.29	0.19	0.80
	10^5^	28.06	0.67	2.38	27.71	0.26	0.93
BNoV	10^7^	14.49	0.09	0.64	14.58	0.12	0.81
	10^6^	19.98	0.05	0.25	19.89	0.17	0.84
	10^5^	24.28	0.46	1.91	23.98	0.33	1.39
BCoV	10^7^	17.32	0.22	1.29	17.19	0.09	0.50
	10^6^	20.51	0.46	2.27	20.55	0.03	0.15
	10^5^	24.02	0.03	0.11	23.92	0.07	0.29
BRV	10^7^	17.83	0.01	0.01	17.75	0.06	0.32
	10^6^	21.37	0.07	0.03	21.31	0.07	0.31
	10^5^	24.85	0.06	0.02	24.93	0.06	0.23
BVDV	10^7^	20.71	0.04	0.17	20.71	0.01	0.06
	10^6^	24.08	0.01	0.02	24.05	0.03	0.10
	10^5^	27.49	0.01	0.03	27.44	0.05	0.20

### 3.6 Calf fecal sample testing

The applicability of multiplex real-time PCR was evaluated in this study by testing 295 calf diarrhea samples (Table 5). Among these diarrhea samples, one was positive for BToV, 18 were positive for BEV, two were positive for BNoV, four were positive for BCoV, 32 were positive for BRV, and six were positive for BVDV. Therefore, the positivity rates for BToV, BEV, BNoV, BCoV, BRV, and BVDV were determined to be 0.34%, 6.44%, 0.68%, 1.36%, 10.85%, and 2.03%, respectively.

**Table 5:** Multiplex real-time fluorescence quantitative PCR testing of calves with diarrhea in Guangdong, China.

	**BToV**	**BEV**	**BNoV**	**BCoV**	**BRV**	**BVDV**
Guangzhou A	1/31	1/31	0/31	1/31	0/31	0/31
Guangzhou B	0/51	0/51	0/51	2/51	27/51	4/51
Foshan	0/36	12/36	1/36	0/36	5/36	0/36
Meizhou	0/72	1/72	0/72	0/72	0/72	0/72
Huizhou	0/33	4/33	1/33	1/33	0/33	1/33
Yangjiang	0/48	0/48	0/48	0/48	0/48	1/48
Heyuan	0/24	0/24	0/24	0/24	0/24	0/24
Total amount	1/295	18/295	2/295	4/295	32/295	6/295

## 4 Discussion

Calf diarrhea poses a significant economic burden on the global cattle industry, which has primarily been attributed to viral infections that have a particularly severe impact on calves (Santman-Berends et al., 2015; Pinior et al., 2019). While cases of acute death after viral infection are rare, these viruses have the characteristics of prolonged infection periods, rapid transmission, and wide dissemination, often leading to large-scale epidemics and substantial economic losses.

Infections with BEV, BToV, BNoV, BCoV, BRV, and BVDV can result in diarrhea in cattle and are often accompanied by co-infection with other viruses, parasites, and bacteria (Brunauer et al., 2021; Zhu et al., 2022). This can lead to severe diarrhea, bloody stools, dehydration, and even death in affected cattle. Moreover, surviving cattle may experience growth retardation or stagnation, which can significantly affect their performance. At present, there are no commercial vaccines against the other five viruses except BVDV in China. In particular, the understanding of BEV, BToV, and BNoV, which have only been detected in China in recent years, remains limited (Guo et al., 2018; Shi et al., 2019, 2020). Nonetheless, despite the isolation of BCoV, BRV, and BVDV as early as the twenteeth century, and the continued research and development of vaccines, their prevalence rates have remained high (Gomez et al., 2017; Zhu et al., 2022). To effectively prevent and control these diseases, it is necessary to achieve long-term detection of emerging epidemics, thereby allowing the isolation and elimination of diseased cattle to reduce economic losses. Therefore, it is critical to establish a rapid, stable, and sensitive method for the prevention and control of these bovine viruses.

At present, multiple PCR and qPCR methods have been established to detect calf diarrhea-causing viruses (Thanthrige-Don et al., 2018; Goto et al., 2020), however, compared with these methods, our method introduced a new pathogen, BNoV, and further enriched the multiple detection method for calf diarrhea-causing viruses. Although BNoV is rarely reported as an important severe diarrhea-causing pathogen in calves, it still warrants attention. The multiplex real-time fluorescence-based quantitative PCR established in this study allows the simultaneous detection of six viruses: BEV, BToV, BNoV, BCoV, BRV, and BVDV. This method is essentially joint application of real-time fluorescent quantitative PCR. This method was designed because of the limited channels of current fluorescence quantitative PCR instrument on the market. Because of this instrumental limitation, we aimed to establish a system of simultaneous real-time fluorescent quantitative PCR method for six targets–a complicated task. Therefore, we adopted two multiple combined composite detection methods of the real-time fluorescent quantitative PCR, each system testing three targets, to ensure simultaneously amplification and fluorescent signal acquisition, effectively testing six kinds of viruses simultaneously.

The reaction procedure established in this study, which utilizes two reaction systems, can detect the aforementioned viruses within just 1 h, providing a relatively fast detection strategy. Moreover, compared with single-pathogen detection methods, this strategy is considered easy to operate and efficient. The detection limits of this method for BNoV, BEV, BToV, BRV, BCoV, and BVDV standard plasmids were determined to be 1.91 copies/μL, 96.0 copies/μL, 12.8 copies/μL, 16.4 copies/μL, 18.2 copies/μL, and 65.3 copies/μL, respectively. Further, the sensitivity of this method is 10–10000 times higher than that of other conventional RT-PCR assays (Zhou et al., 2017; Yang et al., 2020). The amplification efficiency of the standard plasmid was found to be ~90%−110%. Additionally, the linear expression R^2^ for all six standard curves was ≥ 0.980, indicating a strong linear relationship. Finally, the CVs of the intra- and inter-assay replicates of the standard plasmid were <3%, indicating that the method established in this study had good repeatability and stability. Institutes that detect viruses frequently encounter RNA viruses, and for actual detection RNA has to be transcribed to cDNA before testing. However, in the process of establishing a detection method based on plasmid DNA as a template, the inability to fully demonstrate the sensitivity of the assay to the sample is an important shortcoming. Accordingly, a substantial number of clinical samples need to be collected to verify the amplification efficiency.

Using this established method, we subsequently tested 295 fecal and anal swabs collected from calves with diarrhea in Guangdong, China. The positive rates of BToV, BEV, BNoV, BCoV, BRV, and BVDV in these samples were 0.34%, 6.10%, 0.68%, 1.36%, 10.85%, and 2.03%, respectively. Notably, the positive rates of BRV and BEV were the highest among the tested samples. Additionally, this was the first study to detect BToV and BNoV in Guangdong, indicating the prevalence of these viruses in this region. Although the detection rate of BToV and BNoV was not high, they still warrant attention as an emerging infectious disease in China. In general, the positive detection rates of viruses was not high. Bacterial and parasitic infections and indigestion-induced diarrhea were found in some of the other fecal samples that tested negative for diarrhea, indicating that there was no single factor causing diarrhea in calves in Guangdong Province. Further large-scale sampling is required to investigate the prevalence of calf diarrhea in Guangdong Province, China.

## 5 Conclusion

In summary, this study established a rapid, convenient, highly sensitive, and stable method for the detection of BEV, BToV, BNoV, BCoV, BRV, and BVDV using two reaction systems with the same reaction procedure. BRV and BEV were determined to be the most prevalent viruses in 295 calf samples collected in Guangdong, China. Moreover, we preliminarily confirmed the occurrence of BToV and BNoV infection in calves in this region using the method established in this study. Nonetheless, large-scale sampling is required to further investigate the prevalence of these two viruses in Guangdong, China. Ultimately, this detection method can be used to improve the prevention and control of diarrhea-associated viral infections in calves.

## Data availability statement

The original contributions presented in the study are included in the article/supplementary material, further inquiries can be directed to the corresponding authors.

## Author contributions

WM: Writing—original draft, Writing—review & editing. ZC: Software, Writing—original draft. QJ: Data curation, Writing—original draft. JC: Supervision, Writing—original draft. XG: Validation, Writing—original draft. ZM: Writing—original draft. KJ: Funding acquisition, Resources, Writing—review & editing. SL: Funding acquisition, Resources, Writing—review & editing.
